# Early Psychosis Service User Views on Digital Technology: Qualitative Analysis

**DOI:** 10.2196/10091

**Published:** 2018-10-31

**Authors:** Sandra Bucci, Rohan Morris, Katherine Berry, Natalie Berry, Gillian Haddock, Christine Barrowclough, Shôn Lewis, Dawn Edge

**Affiliations:** 1 Manchester Academic Health Science Centre Division of Psychology and Mental Health, School of Health Sciences University of Manchester Manchester United Kingdom; 2 Greater Manchester Mental Health NHS Foundation Trust Manchester United Kingdom

**Keywords:** qualitative, psychosis, framework analysis, digital health, mHealth, mobile phone

## Abstract

**Background:**

Digital technology has the potential to improve outcomes for people with psychosis. However, to date, research has largely ignored service user views on digital health interventions (DHIs).

**Objective:**

The objective of our study was to explore early psychosis service users’ subjective views on DHIs.

**Methods:**

Framework analysis was undertaken with data obtained from 21 semistructured interviews with people registered with early intervention for psychosis services. Robust measures were used to develop a stable framework, including member checking, triangulation, independent verification of themes, and consensus meetings.

**Results:**

The following 4 themes were established *a priori*: acceptability of technology in psychosis and mental health; technology increasing access to and augmenting mental health support; barriers to adopting DHIs; and concerns about management of data protection, privacy, risk, and security of information. The following 2 themes were generated *a posteriori*: blending DHIs with face-to-face treatment and empowerment, control, and choice. DHIs were also viewed as potentially destigmatizing, overcoming barriers faced in traditional service settings, facilitating communication, and empowering service users to take active control of their health care.

**Conclusions:**

In the first study of its kind, early psychosis service users’ were largely positive about the potential use of DHIs supporting and managing mental health. Overall, service users felt that DHIs were a progressive, modern, and relevant platform for health care delivery. Concerns were expressed around privacy and data security and practical barriers inherent within DHIs, all of which require further attention. Future research should explore whether findings transfer to other service user groups, other technology delivery formats, and across a range of treatment modalities.

## Introduction

Integration of technology into health services is becoming commonplace, primarily owing to recent developments in hardware and connectivity. Along with facilitating direct contact between service users and clinicians [1,2] digital monitoring and health intervention tools have been recently applied in the treatment of psychosis with promising effects [3-8]. Given the inverse relationship between the age and use of digital health technology [9], computer literacy [10,11], and mobile phone ownership or use [12-14], mobile health (mHealth) systems may be particularly advantageous when applied to an early psychosis population vulnerable to relapse [15]. Levels of technology use in psychosis are similar to that in the general population [12,3], and people with psychosis express favorable attitudes toward digital health interventions (DHIs) and self-management systems [3].

Despite potential advantages of technology integration into mental health care [16], few investigations have focused on service users’ subjective views of digital systems [17]. Palmier-Claus et al [18] explored the views of 24 people with psychosis about ClinTouch self-monitoring of symptoms with a smartphone app. The following 3 key themes were identified: usability and familiarity with the technology, acceptability and integration of technology into daily life, and perceived impact of technology on health care. Another qualitative study with individuals experiencing psychosis reported that using Web-based resources to access mental health-related information was commonplace with many participants expressing positive attitudes toward the potential of mental health apps for self-care [19]. An in-depth understanding of the views of early psychosis service users, however, is yet to be reported.

Key United Kingdom (UK) policies have set clear recommendations regarding a closer involvement of service users in the development of digital innovations and enabling stakeholders’ engagement with digitally enabled services (eg, National Health Service, NHS, Five Year Forward View [20]). Evidence suggests that involving service users in intervention development is associated with high levels of engagement with DHIs more generally [21], highlighting the need to examine service users’ perspectives on DHIs.

This study aimed to engage with early psychosis service users to gain an in-depth understanding of their perspectives and attitudes toward DHIs. The study is part of a larger program of work to codevelop and test a DHI for early psychosis [22]. To our knowledge, this is the first study to examine qualitatively early psychosis service users’ views about a DHI delivered via a smartphone app.

## Methods

### Overview

The study was a collaborative partnership involving clinical academics, clinicians, and early psychosis service users who were all involved in the study design, topic guide development, and analysis and interpretation of data.

### Study Design

This was a qualitative investigation nested within a broader research program concerned with development, feasibility, and acceptability of a theory-informed smartphone app for early psychosis, *Actissist* [22]. Data were gathered from semistructured interviews (N=21). The study was funded by the Medical Research Council, UK, and received ethical approval from the National Research Ethics Committee West Midlands–South Birmingham (14/WM/0118).

### Participants

The purpose of recruitment was to identify participants who could provide insight into the phenomenon being studied rather than achieving a random or representative sample of the population. Therefore, we used a systematic, nonprobabilistic sampling approach to recruit a purposive sample of service users registered with early intervention for psychosis services (EISs) across the North West of England. EISs are multidisciplinary community mental health services that provide psychosocial and pharmacological treatment and support to people aged 14-65 years in the first 3 years of their initial episode of psychosis. Recruitment was over a 22-week period. Study exclusion criteria were kept to a minimum to include a diverse range of views and experiences. Eligibility criteria were as follows: ability to provide informed consent; registration with EISs; English speaking; and consent to record interviews digitally and publication of deidentified data.

### Procedures

A researcher contacted team managers and gave presentations about the study at service meetings. Subsequently, clinicians identified potential participants and gained consent to contact. A researcher met with participants either in their own homes or at convenient locations. Following consent, semistructured interviews were conducted using a topic guide (available on request) developed for the study based on review of the literature and Smith’s [23] guidelines for constructing the semistructured interview schedule. The topic guide was refined in collaboration with an expert reference group convened for the broader *Actissist* trial [22]. Open-ended questions were designed to explore the following broad areas: participants’ use of technology generally; views about receiving health care and psychological support via smartphone technology; whether mental health apps make sense in the context of service users’ daily lives; incentives and barriers to use; equity and ethics; privacy concerns; and participants’ recommendations and requirements for a mental health app. All interviews were conducted by RM who was trained by an experienced clinician and academic (SB) and qualitative methodologist (DE). The order in which topics emerged was influenced by the topic guide but was not exclusively driven by it. Interviews were conducted as part of an iterative and inductive process of data collection and analysis [24], that is, as understanding of relevant issues developed, the topic guide was altered to focus interviews on emerging themes, thus allowing the data to drive development of relevant questions; for example, participants spontaneously spoke about the importance of personification of a mental health app, resulting in the inclusion of a related question in the topic guide. With each additional issue raised, we recontacted participants interviewed prior to the addition of new items to elicit their views regarding such issues. Interviews were digitally recorded and transcribed verbatim.

### Data Analysis and Framework Development

Data were analyzed using a framework analysis approach [25]. While sharing common features with other qualitative approaches (eg, thematic analysis), framework methodology makes explicit a visible, systematic process that allows for the inclusion of both *a priori* and emergent concepts. With the help of a service user expert group, we developed a list of important topics we wished to seek views about prior to developing the framework. With these topics in mind, questions for the topic guide were developed and subsequently informed the framework’s *a priori* themes. Specifically, we explored people’s previous and current satisfaction with using mental health services; perceptions of DHIs and the ability of technology to facilitate symptom monitoring and support self-management; incentives and barriers to use and implementation of mHealth tools; experiences of using technology to support mental health; and the impact of DHIs on disclosure of risk and governance issues. Therefore, the following 4 themes were established *a priori*: acceptability of technology in mental health; technology’s ability to increase access to, and augment, mental health support; barriers to adopting digital solutions; and data protection, privacy, and security of information. At the same time *a priori* topics were being explored, other themes, which participants’ spontaneously described, emerged from the data. The following 2 themes emerged from the data *a posteriori:* blending DHIs with face-to-face treatment and empowerment, control, and choice.

Although nonlinear and often condensed, data analysis involved the following key stages: (1) familiarization with the data: listening to recordings, reading and rereading transcripts and making analytical notes; (2) coding the data: combination of deductive (using predefined codes based on specific research questions) and inductive approaches (using “open coding” to identify any emergent, possibly relevant information); and (3) developing a thematic framework: we developed an initial framework by comparing codes assigned to the data after independently coding several transcripts before agreeing on the set of codes to be assigned to subsequent transcripts. Subsequent framework iterations were shared with the members of the wider research team and participants themselves (“member checking”). We then coded remaining transcripts into the framework and constantly compared new data with the framework. Data were then interpreted and summarized, new codes generated, redundant codes deleted, and overlapping codes merged; (4) indexing: the framework was applied to the dataset; (5) charting: a framework matrix for each emergent category across the whole dataset using illustrative quotations was developed using QSR International’s NVivo 10 Software data management software; and (6) mapping and interpretation: emergent (*a posteriori*) and *a priori* characteristics of the data were identified and connections between categories “mapped,” facilitating exploration of relationships (similarities and differences) and theoretical concepts and generation of typologies**.**

We took a number of additional steps to enhance the study’s methodological rigor and to minimize researcher bias. Specifically, SB and DE scrutinized interviews and provided feedback and training to the interviewer to minimize any tendency to lead participants; a selection of transcripts was coded independently by authors GH and KB (who were independent of framework development), providing triangulation of analysis and independent verification; framework refinement and development of the analytical matrix was undertaken by all authors. Regular consensus meetings were held until a stable framework emerged. Participant feedback on the framework and subsequent findings (participant verification) were sought from study participants. Data collection ceased when no further themes were advanced (ie, data saturation [26]).

## Results

### Participant Characteristics

Interviews lasted from 39 to 78 minutes. Uptake of study participation was high; 88% of service users that we approached consented to take part in the study. Participants had a mean age of 26 years (SD 5.14, range 16-34) and a mean length of 22 months of EIS involvement. Just over half of participants were female (11/21, 52%), were in full-time employment, education, or training (11/21, 52%), and living with family members, partners, or others (13/21, 62%).

Table 1 summarizes the use of technology and potential barriers of using DHIs across the sample. All participants (21/21, 100%) used the internet primarily for social networking (12/21, 57%), followed by video and audio streaming (9/21, 43%) and research or studying (9/21, 43%). All except one participant owned a mobile phone (20/21, 95%) with the majority owning smartphones (18/21; 90%). All participants (21/21, 100%) had previously used smartphone apps. Two-thirds (12/18, 67%) of the sample reported using apps for health purposes, half (9/18, 50%) for social networking, and one-third (7/18, 39%) for gaming purposes. A third (n=7/21, 33%) of participants reported literacy difficulties. However, these participants reported finding information accessible on a smartphone more accessible than paper-based approaches.

**Figure 1:**
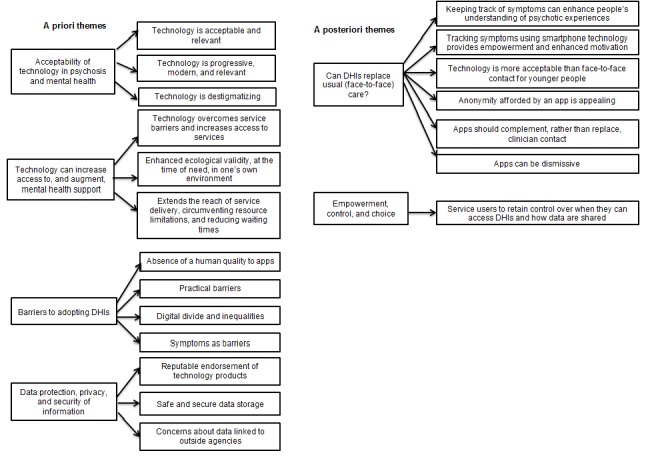
Summary of the framework. DHI: digital health intervention.

The framework is summarized in Figure 1 and elaborated below, as evidenced by key quotations embedded within the text.

### Theme 1: Acceptability of Technology in Psychosis and Mental Health

There was a complete agreement across participants (n=21) that mobile technology is an acceptable and relevant way to gather information about, and access support for, mental health problems. Generally speaking, the idea of using smartphones to seek help was viewed as just as acceptable as traditional methods:

I do think that [technology] is really good cause it’s going to be accessible to people that will need the help. Some people don’t always want to speak outwards. It would be much easier on an app where I could take it with me anywhere at anytime and open it up and record how I am doing....

Participant 15

**Table 1:** Reasons for using the internet and smartphones.

Type of use reported		n (%)
**Internet use^a^**		
	Social networking, blogging	12 (57)
	Video, audio streaming	9 (43)
	Research, studying	9 (43)
	Email	6 (29)
	Gaming	5 (24)
	News	5 (24)
	Web-based banking	1 (5)
	Self-help websites	1 (5)
	Art tools (browser-based app)	1 (5)
**Smartphone apps use^b^**		
	Health purposes (physical & mental)	12 (67)
	Social networking	9 (50)
	Gaming	7 (39)
	Art (including photography)	4 (22)
	News	4 (22)
	Appointment reminders or calendar	3 (17)
	Shopping	3 (17)
	Banking	2 (11)
	E-books	2 (11)
	Global positioning system	1 (6)
	Television guide	1 (6)

^a^n=21.

^b^n=18.

Technology was viewed as a good way of accessing help and support when needed because participants reported often feeling restricted by traditional face-to-face service provision:

It’s not like a GP [general practioner] where you’ve gotta go up the road and then speak to him. [using technology] You can easily sit in your own home and read through the app...when I’m going to a GP...I’m silent.

Participant 14

Nearly half of the participants (10/21, 48%) held the view that technology is progressive, modern, and relevant and that mental health apps reflect a good way of “moving with the times,” which is more in keeping with how young people communicate on a daily basis. Making this link between day-to-day communication styles and engaging with health services that reflects current methods young people use to interact with each other was viewed as positive and progressive development of mental health care:

I’m very good on computers so it’s easier for me to type than it is for me to speak to someone. People these days are quite up on apps and stuff...

Participant 10

Participants expressed the view that technology has the capacity to be destigmatizing. Smartphones, as opposed to mental health settings, were viewed as inherently normalizing because majority of people use and carry this technology:

You've got these people turning up at your front door and they’ve got their health things on round their necks...you might as well be wearing a sign really. One woman took me to [retail store] and told me she goes there with a lot of other service users. That made me incredibly anxious because I thought other people that work here are gonna know what her job is, whereas everyone uses an app these days innit? It’s normal now.

Participant 3

However, not all participants shared this view; others described feeling embarrassed or uncomfortable while using a mental health app in front of other people:

If the app is asking you to pull it out every time you’re in a social situation, it gets embarrassing and that can add to the anxiety you feel in a social situation.

Participant 13

### Theme 2: Technology Can Increase Access to and Augment Mental Health Support

In many instances, participants expressed the view that apps could overcome barriers to traditional service set-up and, in particular, increase access to treatment and services because the use of technology does not depend on workers being available at specific times. Support could be accessed in one’s naturalistic environment at the point of need and was therefore viewed as having high ecological validity:

I’ve already had CBT [cognitive behavior therapy]...I think I would have been more successful with it if I had something like this because there’s lots of elements of it [CBT] where you’re supposed to be keeping diaries of your moods and if you’re low...If there’s nothing to prompt you other than yourself, it’s very hard to motivate yourself and then you might find yourself coming to your weekly appointment realizing you haven’t bothered to fill in any of this stuff out for the last 4 days and just trying to make it up on the spot.

Participant 16

Participants’ accounts suggested that technology could extend the reach of service delivery, circumventing resource limitations and reducing waiting times:

I think it would cut down on time that people will have to wait to see a health professional...some people wouldn’t need to see a professional face-to-face, they might just be able to deal with their issues via the app.

Participant 11

Furthermore, a few participants (n=6) commented on the fact that secondary or related symptoms of psychosis (eg, sleep problems and social withdrawal) or negative beliefs about technology itself causing harm can make it difficult to attend traditional clinics:

If you are someone that’s awake all night and you sleep all day or you struggle to leave the house then you’re going to struggle with face-to-face [contact].

Participant 8

### Theme 3: Blending Digital Health Interventions With Face-to-Face Treatment

There were mixed views on whether mental health apps could be used as a stand-alone intervention or whether it should be clinician supported. In general, participants felt that DHIs offered more benefits compared with face-to-face contact. Participants were positive about the ability of smartphone technology to keep a track of their symptoms and experiences. Many thought that this ability would actually enhance their understanding of psychotic experiences.

I think it would be a great help because people would be able to see the warning signs very early on and go ‘hang on a second, this isn’t right, what do I need to do to help myself’

Participant 16

Additionally, DHIs seemed to give people space to understand their experiences for themselves:

Sometimes it’s better when you’re on your own and you get to create your own opinions on how you’re feeling and understanding how you feel instead of being told how you feel.

Participant 17

Participants who had used symptom-monitoring apps in the past felt that the ability to track symptoms using smartphone technology facilitated feelings of empowerment and enhanced motivation in a way traditional health care face-to-face delivery could not:

You learn a bit more about yourself and how you're actually feeling at that moment...it helped motivate me to improve.

Participant 11

Some younger participants identified that because they have grown up with technology, they find digital means of communication easier than face-to-face methods:

For me personally, I’d rather talk online. You know, if people aren’t going to talk to someone [face-to-face] then with an app they can still deal with their problems.

Participant 9

It’s easier for me to type than speak cos I was brought up with computers.

Participant 16

The fact that an app is anonymous appealed to some because direct clinician contact can reinforce people’s sense of guilt or failure if they have not completed therapy tasks or complied with medication:

You don’t feel guilty if you haven’t done your homework.

Participant 6

In addition, although many participants described the perceived value of talking to a clinician face-to-face, others did not share this opinion; for example, some participants said that they would feel much more comfortable using an app to support their mental health problems rather than talking to a member of their care team:

I think there’s one side of it that could really work which is kind of like the exercises...itcould talk you through them, and actually not being in front of a person, you might do them more truthfully.

Participant 7

A few participants, however, felt that apps should be used as an adjunct to, rather than a replacement for, direct clinician contact so that DHIs complement rather than replace clinician-supported care:

I think it would not replace one-to one talking therapy but I think there are aspects of [an intervention] which could be put into an app, which you could access in-between sessions of talking one-to-one with someone.

Participant 7

Some participants noted using an app, rather than seeing a clinician, might feel dismissive as though they are not worthy of a clinician or therapist. There was cynicism among some participants, albeit the minority, that technology adoption across health services was a cost-cutting exercise, which would ultimately be detrimental to the health care people receive. If used in conjunction with traditional clinician-delivered care, participants thought that apps could be helpful. However, they wanted control over how they use the app and with whom the information is shared.

There’s some things I don’t share with my [clinician] that I don’t want him to know yet and for him to be able to find it in a diary, I wanna be able to say‘actuallycan we skip that day? It’s a really personalday’

Participant 9

Of note, other participants said that if they knew that their information was shared with their care team, they might interact differently with the app, for example, by being a “*little less honest*,” as evidenced by Participant 21:

I would describe my symptoms as not as bad because I wouldn’t want my [clinician] worrying or thinking that the treatment wasn’t working.

Participant 21

Although some people thought that sharing risk information would compromise trust and might affect the way they interact with digital technology (“I would be more careful and less experimental,” Participant 8), many participants thought that their mental health was something private. On the whole, participants thought that reporting risk to their care teams would be advantageous and potentially life-saving, leading to better focused care.

### Theme 4: Empowerment, Control, and Choice

The majority of interviewees believed that smartphone technology could facilitate a sense of personal ownership and control over their health care:

If I had an app I would have kept on top of [my mental health] a lot better. I don’t like scenarios where I feel my mental health is dictating my life and that is all that my life is, and that’s how it feels when you're going to appointments all the time...an app would be just kind of enabling people to be empowered themselves, to take their care into their own hands. I think there’s a habit for people to be quite passive in their care. They think ‘the Dr. knows best’

Participant 2

A common belief among interviewees was that people should be given options about the health care they receive and treatment choice, which may differ at times; for example, some interviewees highlighted a stepped-care pathway, wherein an individual might choose to use an app exclusively at a specific point in time in the course of their recovery but incorporate human contact at other times:

If you’re at an all-time low then you might think ‘right, I need to actually speak to someone’. If it’s sort of like creeping on and you’re just feeling a bit, just crying, then the app would be handy.

Participant 5

### Theme 5: Barriers to Adopting Digital Health Interventions

A number of barriers to using technology-related mental health tools were identified. Some interviewees described the absence of a human quality and lack of emotional reassurance and feedback offered by apps problematic because DHIs provide limited opportunities to connect and interact at an emotional and interpersonal level:

Talking to somebody is very personal. You can get their instant reaction, their emotions and everything. When you're opening up it’s crucial that you have somebody there to reassure you.

Participant 20

If you are talking to a machine, you know you’re talking to a machine, so if it tries to pretend it’s a human, even if you’re allowing yourself to go along...you are being degraded in a way.

Participant 12

Practical barriers, such as forgetting to turn on or charge the phone and losing or breaking the phone, could impact engagement with digital tools. Furthermore, the concept of the “digital divide” (inequalities with regard to access to, use of, or provision for information and communication technologies) was noted by some participants who highlighted that some people do not have access to smartphones, thus limiting their ability to access DHIs. Indeed, even participants with access to smartphones stated that poor data allowance would prevent them from using the technology:

On my phone I only get like 1 GB out of it which runs out quick.

Participant 5

### Theme 6: Data Protection, Privacy, and Security of Information

About two-thirds (16/21, 76%) of participants expressed concerns about data protection and information governance. However, participants stated that their fears about information safety could be allayed if services reassured them about data safety. Many participants said that endorsement of a DHI by a valid institution (eg, university, health service, or respected, well known mental health charity) would be sufficiently reassuring and would increase DHI uptake. However, a minority of interviewees said they would prefer endorsement by individuals (eg, care co-ordinator and doctor) rather than by organizations because “organizations have hidden agendas” (Participant 13). Alternatively, a strong relationship between a service user and staff member working in a service would be sufficiently reassuring for some interviewees.

I trust the early intervention team and people associated with it, so I would be fairly confident that it would be secure, if they said so.

Participant 7

Some participants identified that data stored locally on the smartphone and on a server needs to be safe, secure, and private and that, ideally, data should be “locked” on the phone. On the whole, participants did not report concerns about clinical services gaining access to their data per se. Rather, concerns were expressed about data being linked to outside agencies (eg, commercial search engines, iCloud, and social networking sites):

Storing it in iCloud wouldn’t be acceptable,storing it by email, sending it in email that is unencrypted isn’t the greatest way to share data. Those kinds of things should be addressed particularly as it is mental health and mental health has a strong taboo in society...Ifit was leaked,it would be disastrous for the people involved.

Participant 15

## Discussion

### Overview

In light of the inevitable adoption of a worldwide digital health service, it is surprising that this is the first qualitative study to examine early psychosis service users’ perspectives on digital technology use for health care needs. We found that in an early psychosis sample, DHIs were just as acceptable as traditional methods and preferable in some instances for seeking information about, and support for, mental health problems.

### Principal Findings

Despite concerns around privacy and data security and some practical barriers inherent within digital platform systems, early psychosis participants’ views were largely positive about the potential use of DHIs in supporting and managing mental health difficulties. These findings are largely consistent with the broader literature across a range of mental health problems [27-29] and reflect some views of carers for early service users [30]. Overall, 6 themes were evident. First, participants felt that apps could enhance services’ accessibility by providing a platform for service users to be open and honest in a way they might not be able to be in traditional clinic-based appointments. These findings support previous assertions by clinicians that the faceless and anonymous nature of DHIs may allow service users to be more open and honest about their experiences [31] and provide more access to Web-based information rather than face-to-face meetings with a clinician [19]. These findings also echo views from a Spanish sample of outpatients with established schizophrenia, who also felt that digital health technology could improve contact with clinicians, affording the chance to have greater contact with health professionals [32]. Technology was not only viewed as a progressive, modern, and relevant platform for health care but also inherently destigmatizing. Perceived stigma is a key barrier to engagement with mental health care services [33]; provision of intervention delivery options that are destigmatizing is therefore warranted. Second, participants reported that DHIs could increase access to, and augment, support by extending the reach of services to one’s naturalistic environment, at the point of need, potentially circumventing lengthy waiting times. Although the national (UK) guidelines recommend the provision of psychological therapies for early psychosis [34], factors such as a limited number of trained clinicians, service cost, and resource pressures mean that many people who could benefit are often unable to receive timely access to evidence-based treatment [35]. Our findings suggest that service users find implementation of DHIs in early psychosis services an acceptable avenue for health care provision. This echoes the findings of the study conducted by Aref-Adib and colleagues (2016), in which semistructured interviews with 22 people with psychosis accessing secondary care services. Participants reported finding Web-based information more accessible across space and time compared with receiving information and support from clinicians. In the context of bipolar disorder, people have also reported that apps facilitate clinician understanding of the user’s experiences of symptoms, encourage shared decision making about treatment, improve service user-clinician communication [27], and enhance clinical care, making time spent with clinicians more efficient. People with more established psychosis have also emphasized how DHIs can help clinicians gain insights into service users’ mental states, potentially leading to earlier and more effective intervention because service users do not need to rely on retrospectively recollecting symptoms when using DHIs capable of capturing experiences in the moment [18]. These views are similarly highlighted by carers for early psychosis service users, who see the value in DHIs facilitating communication with service providers, particularly during times of social withdrawal [30].

Views about whether DHIs could replace face-to-face contact were mixed. Some participants, particularly those who find the clinic environment threatening, indicated that DHIs could indeed replace clinician contact. Creating a safe distance from a clinician facilitates openness and honesty about distressing experiences and facilitates empowerment. Indeed, recent interviews with individuals who had received the *Actissist* app [22] revealed that some participants felt more comfortable in being open and honest compared with face-to-face support options. Previous studies have also highlighted that both clinicians and service users view DHIs for people with severe mental health problems as empowering owing to the transfer of control and power from the clinician to the service user and the opportunity for service users to take meaningful and active control over their health care needs [13,36]; for example, in an established psychosis sample, Aref-Abid and colleagues (2016) found that the act of independently seeking information related to one’s health online and the understanding and knowledge gained as a result of seeking information online was closely linked to feelings of control and empowerment. This finding was also supported in a meta-synthesis review of experiences of computer-delivered therapy for people with depression and anxiety, whereby participants referred to the empowering nature of computerized therapy [36]. In contrast, traditional service settings have been viewed by some service users as disempowering owing to lack of shared decision making and involvement in developing and monitoring treatment and care plans [37,38]. Findings highlight the potential utilization of DHIs for providing early psychosis service users the control and choice over treatment options and support such policy documents as the NHS Constitution Pledge and Five Year Forward View to improve the provision of shared decision making and promotion of service user choice.

Despite the acceptability of DHIs highlighted in this study, some participants viewed apps as potentially invalidating. Digital tools should complement, rather than replace, clinician contact. These findings support conclusions drawn by previous qualitative interviews with psychosis service users who used a symptom-monitoring app; they described the need for clinician involvement and the potential benefits of mental health apps for facilitating service user-clinician communication [18]. Additionally, views expressed in this study mirror those expressed by secondary mental health care staff who believed that an app should never be offered as a stand-alone replacement for face-to-face support options [31]. Participants argued for choice about how DHIs could be used in the health care setting.

Furthermore, data security, safety, and risk require careful consideration and management. This concern is not limited to early psychosis. Similar concerns have been raised in the general population; for example, participants drawn from a large community sample in Australia expressed concerns around privacy issues related to mHealth programs and described the importance of Web-based security, anonymity, and privacy [28]. Privacy and security concerns have also been raised among people with bipolar disorder [27] with particular concerns reported around handset access and secure storage of data in apps. Carers for early psychosis service users have also expressed the importance of safeguard measures, specifically in terms of the professional’s role in how DHI platforms are used [30]. We found that participant concerns around this issue could be allayed if a trusted source endorsed the system. However, recent reviews of publically available smartphone apps revealed that less than a quarter of those available for bipolar disorder included a privacy policy [39] and less than 10% of those available for social anxiety provided organization information [40]. This contrast between current information provided on publically available mental health smartphone apps and the preference of service users for DHIs from trusted sources suggests that content information currently available may not be sufficient to alleviate service user privacy concerns, thus potentially negatively impacting engagement. Future developers must ensure that clear and explicit statements regarding privacy and organizational sources are made available.

On the whole, DHIs were viewed as destigmatizing. The potential of DHIs to enhance service user power, control, and choice over the pathway of care reflected the desire for service user-centered approaches to mental health care, incorporating DHIs that are truly coproduced from the outset [41].

### Strengths and Limitations

This is the first study to explore early psychosis service users’ views on use of digital solutions for health care. Service users have highlighted important factors that researchers and technical developers need to consider when designing and building digital systems in mental health. Our methodological and analytical approaches were rigorous. We allowed the interview schedule to drive development of relevant interview questions by regularly reviewing it and the data gathered, allowing in-depth examination of participant-driven relevant issues. Formal processes to ensure credibility of our findings, including independent peer verification and member checking processes, were exhaustive, ensuring that all participants’ views were thoroughly considered as new themes emerged.

Findings need to be considered in light of the study limitations. Interviews were with an early psychosis group, who, based on the mean age of our sample and smartphone ownership rates, are considered “digital natives,” rendering the sample inherently familiar with smartphone technology [42]. Nevertheless, the sample was a mix of relatively young men and women who were reflective of the early intervention sample we sought to examine. Because most participants were in some form of employment or training, this might be less reflective of other early intervention samples. Participants were recruited in the context of a larger DHI trial and may have already held favorable views toward technology use. However, examination of our findings suggests that service users were well versed in the pros and cons of DHIs for mental health. Previous experience with mental health apps and related products, negative experiences with traditional mental health services, and socially desirable responses during interviews might have influenced participants’ expressed views.

### Implications and Recommendations

Until now, early psychosis service users’ views on DHIs for mental health care have not been considered. This may be, in part, owing to the fast-paced rate of digital technology adoption and the sense of urgency evident in development of DHIs. This study provides a timely exploration of service user views and highlights the potential facilitators and barriers to adoption that must be considered during DHI development. First, the study highlighted that DHIs were acceptable to service users with early psychosis owing to access via an app being destigmatizing, normalizing, progressive, modern, and relevant. These findings highlight the potential for health care apps to mirror how people currently communicate in their routine day-to-day lives. Nevertheless, DHIs require regular updating to remain relevant. Further consideration must be given to smartphone access and data allowance prior to DHI implementation to minimize digital exclusion. A smartphone loan scheme, supported funding, or discounts for medical use warrant further consideration for DHI service adoption.

Participants placed a significant emphasis on the importance of choice, particularly in relation to whether DHI would be used in conjunction with, or as a replacement for, clinician-delivered care. However, further consideration should also be given to what point in the service user’s recovery journey a DHI might be most useful and when clinician contact might be needed (if at all). Furthermore, the process of shared decision making is important to consider. According to these data, service users would like to be given choices regarding information they share with health professionals. Service user choice around, but not limited to, these issues should remain at the forefront of DHI development and implementation.

These findings also highlight the need for focused consideration of secure data collection and storage and reassurance about this, ensuring that service users are fully informed about governance issues. Finally, future research should explore whether our findings transfer to other service user groups, in a broad context of technology delivery formats, across a range of treatment modalities.

## Abbreviations

CBT: cognitive behavior therapy
DHI: digital health intervention
EIS: early intervention for psychosis service
GP: general practitioner
mHealth: mobile health
UK: United Kingdom
NHS: National Health Service
